# Understanding the Role of Communication Technology in Facilitating Social Connectivity and Addressing Loneliness

**DOI:** 10.1093/geroni/igab046.3546

**Published:** 2021-12-17

**Authors:** George Mois, Jenay Beer, Kerstin Emerson, Tiffany Washington

**Affiliations:** 1 University of Illinois, Champaign, Illinois, United States; 2 University of Georgia, Athens, Georgia, United States

## Abstract

In the United States, two out of five adults report feelings of loneliness. The evolvement of communication technologies presents a promising potential in helping improve social connectivity and address the experience of loneliness. However, the sense of presence (embodiment) users are able to achieve through the technologies can vary depending on their abilities and functions. The present study identified user characteristics associated with an interest to adopt telepresence technologies (e.g., videoconferencing, smart displays, robots) across various levels of embodiment. The data for this study were collected using a Qualtrics survey which was distributed via Amazon Mechanical Turk. The participants recruited for this study were between the ages of 18-78 years old, constituting a total sample size of 384 participants. The data were analyzed using four logistics regression models. The dependent variables aimed to identify participants' interest to adopt telepresence technologies across varying embodiment levels. Across the lifespan older adults were significantly more likely to report lower rates of overall loneliness than young and middle-aged adults. Our findings indicate that those interested in adopting communication technologies with higher levels of embodiment had significantly higher odds of reporting being divorced or widowed (OR=4.12, p<.05), reside in a rural community (OR=2.20, p<.05), and report higher rates of emotional loneliness (OR=1.20, p<.05). Across the four models, there was no significant difference in participants' interest to adopt telepresence technologies. These results suggest that the sense of presence achieved across the various types of communication technologies may help address feelings of loneliness and support healthy aging.

